# Cardiovascular outcomes associated With GLP-1 and dual GLP-1/GIP receptor agonists in patients with type 2 diabetes and inflammatory bowel disease: A retrospective real-world cohort study

**DOI:** 10.1371/journal.pone.0358659

**Published:** 2026-09-24

**Authors:** Muhammad Ali Ibrahim Kazi, Syed Musa Mufarrih, Akanksha Sirohi, Fahad Hasan, Akshay Sharma, Sanmeet Singh, Nargiz Muganlinskaya, Nowreen Haq

**Affiliations:** 1 Department of Internal Medicine, Anne Arundel Medical Center, Annapolis, Maryland, United States of America; 2 Department of Internal Medicine, Aga Khan University, Karachi, Pakistan; 3 Department of Internal Medicine, CMH Lahore Medial College and Institute of Dentistry, Lahore, Pakistan; 4 Department of Gastroenterology, Anne Arundel Medical Center, Annapolis, Maryland, United States of America; 5 Department of Endocrinology, Anne Arundel Medical Center, Annapolis, Maryland, United States of America; University of Diyala College of Medicine, IRAQ

## Abstract

**Introduction:**

Individuals with co-morbid T2D and IBD represent a uniquely high-risk group, facing amplified cardiovascular risk through synergistic inflammatory and metabolic pathways, in whom conventional cardiometabolic strategies (such as dietary and lifestyle changes, weight loss, blood pressure and lipid optimization, metformin therapy, SGLT2 inhibitor therapy) may be insufficient due to the overlap between metabolic disease and chronic inflammatory disease processes. At present, the literature available on the role of incretin-based therapy in the treatment of T2D in patients with co-morbid IBD is limited. We aimed to evaluate the association of GLP-1 receptor agonists and dual GLP-1/GIP receptor agonists with cardiovascular outcomes in individuals with T2DM and IBD. This study is not intended as a direct comparison of the efficacy of GLP-1 agonists and dual GLP-1/GIP receptor agonists in patients with T2D and IBD. Both drug classes were analyzed as a combined incretin-based therapy exposure group to reflect real-world prescribing patterns and maximize statistical power.

**Methods:**

We conducted a retrospective cohort study using the TriNetX global federated research network, identifying adults (≥18 years) with T2D and IBD between 2015–2024. Patients prescribed GLP-1 or dual GLP-1/GIP were compared with non-users following 1:1 propensity score matching on demographics, comorbidities, and medication use. Relative risks (RR) and Hazard Ratios (HR) with 95% confidence intervals (CI) were calculated, with significance defined as p < 0.05.

**Results:**

After matching 16,118 patients per cohort from among a total matched population of 32,236 patients, GLP-1 or GLP-1/GIP receptor agonist therapy was associated with lower hazard ratios across all outcomes, including myocardial infarction (HR 0.701, 95% CI 0.637–0.771), chronic ischemic heart disease (HR 0.936, 95% CI 0.896–0.978), heart failure (HR 0.777, 95% CI 0.737–0.820), atrial fibrillation/flutter (HR 0.838, 95% CI 0.788–0.890), sudden cardiac death (HR 0.694, 95% CI 0.605–0.795), ischemic stroke (HR 0.781, 95% CI 0.707–0.862), deep vein thrombosis (HR 0.642, 95% CI 0.586–0.702), and pulmonary embolism (HR 0.722, 95% CI 0.640–0.815).

**Conclusions:**

In patients with comorbid T2D and IBD, GLP-1 and GLP-1/GIP receptor agonists were associated with significant reductions in ischemic, arrhythmic, and thromboembolic cardiovascular outcomes. These findings highlight the potential role of incretin-based therapies in improving cardiovascular risk management in this high-risk population. However, the present study does not establish the comparative efficacy of the two classes of drugs in this patient population, and the findings may be associational rather than causal. Both drug classes were analyzed as a combined exposure group. Mechanistic explanations proposed in this paper should be considered hypothesis-generating rather than confirmed by the current observational study design.

## 1. Introduction

Cardiovascular disease (CVD) is the leading cause of mortality among individuals with type 2 diabetes mellitus (T2D), accounting for over 50% of deaths in this population globally [1] and remaining a major public health concern in the United States, where more than 37 million adults are affected by T2D [2,3]. The increased cardiovascular risk associated with T2D is multifactorial, driven by chronic hyperglycemia, insulin resistance, dyslipidemia, microvascular injury, and endothelial dysfunction [4,5]. In addition to these metabolic abnormalities, persistent low-grade systemic inflammation has been identified as a key contributor to accelerated atherosclerosis and increased rates of myocardial infarction, heart failure, and stroke in patients with T2D [6].

Inflammatory bowel disease (IBD), which includes Crohn’s disease and ulcerative colitis, is also characterized by chronic systemic inflammation and is increasingly recognized as an independent, non-atherosclerotic cardiovascular risk factor, driven by systemic inflammation rather than traditional lipid-mediated mechanisms [7,8]. Individuals with IBD have significantly higher rates of ischemic heart disease, arrhythmias, myocarditis, and thromboembolic events compared with the general population [9]. In the United States, more than three million individuals are living with IBD, with rising prevalence worldwide [10]. The mechanisms underlying cardiovascular risk in IBD include cytokine-mediated endothelial dysfunction, platelet activation, increased arterial stiffness, and a pro-thrombotic state that persists even during clinical remission [11–13].

When T2D and IBD coexist, cardiovascular risk may be amplified through overlapping inflammatory and metabolic pathways. Shared mechanisms include elevated tumor necrosis factor-alpha (TNF-α), interleukin-6 (IL-6), oxidative stress, and impaired vascular reactivity [14–16]. Despite this elevated risk, patients with coexisting T2D and IBD remain underrepresented in cardiovascular outcome trials, and there are no specific guideline-directed management strategies tailored for this high-risk subgroup [15,17]. This gap underscores the need to evaluate therapeutic options that address both metabolic disease and systemic inflammation.

Glucagon-like peptide-1 receptor agonists (GLP-1) and dual glucose-dependent insulinotropic polypeptide/glucagon-like peptide-1 receptor agonists (GIP/GLP-1) represent an important development in the management of cardiometabolic disease. Randomized controlled trials, including LEADER, SUSTAIN-6, and REWIND, demonstrated that agents such as liraglutide and semaglutide reduce major adverse cardiovascular events (MACE) in patients with T2D at high cardiovascular risk [18–20]. In contrast, cardiovascular outcomes data for tirzepatide are not yet available from peer-reviewed randomized trials; existing evidence is derived from surrogate cardiometabolic improvements. Beyond glucose reduction and weight loss, incretin-based therapies exert cardiovascular benefits through anti-inflammatory effects, improved endothelial function, reduced oxidative stress, and modulation of atherogenesis [21–23]. These mechanisms suggest potential benefit for patients with coexisting T2D and chronic inflammatory disorders such as IBD.

Real-world evidence supports these findings. Large observational studies and registry analyses have demonstrated that GLP-1 use is associated with reduced hospitalization for heart failure, cardiovascular mortality, and all-cause mortality compared with insulin and sulfonylureas [24–26]. In addition to cardiometabolic outcomes, incretin therapy has been associated with improvements in treatment satisfaction, weight-related quality of life, and physical functioning—key considerations in chronic inflammatory disease management [27,28].

Despite these findings, no studies to date have evaluated cardiovascular outcomes associated with GLP-1 or dual GIP/GLP-1 therapy specifically in patients with both T2D and IBD. This represents a critical gap in the literature, as patients with coexisting T2D and IBD experience compounding inflammatory and metabolic insults that may render them uniquely responsive to the cardioprotective and anti-inflammatory properties of incretin-based therapies.

GLP-1 receptor agonists and dual GLP-1/GIP receptor agonists share overlapping anti-inflammatory, endothelial-protective, and cardioprotective mechanisms that are particularly relevant in the setting of chronic immune activation. Given the absence of prior evidence in this population and the relatively recent clinical introduction of dual GLP-1/GIP agonists, we analyzed both drug classes as a combined incretin-based therapy exposure to maximize statistical power and provide an initial characterization of cardiovascular outcomes in this underserved population. This study does not aim to compare the efficacy of GLP-1 versus dual GLP-1/GIP receptor agonists; a dedicated head-to-head analysis is planned as future work.

This remains a clinically important evidence gap given the high burden of cardiovascular complications in this population. Understanding the effects of incretin-based therapy in this dual-diagnosis cohort may help inform risk reduction strategies and support personalized therapeutic approaches.

## 2. Methods

### 2.1. Data source and study design

**Fig 1:**
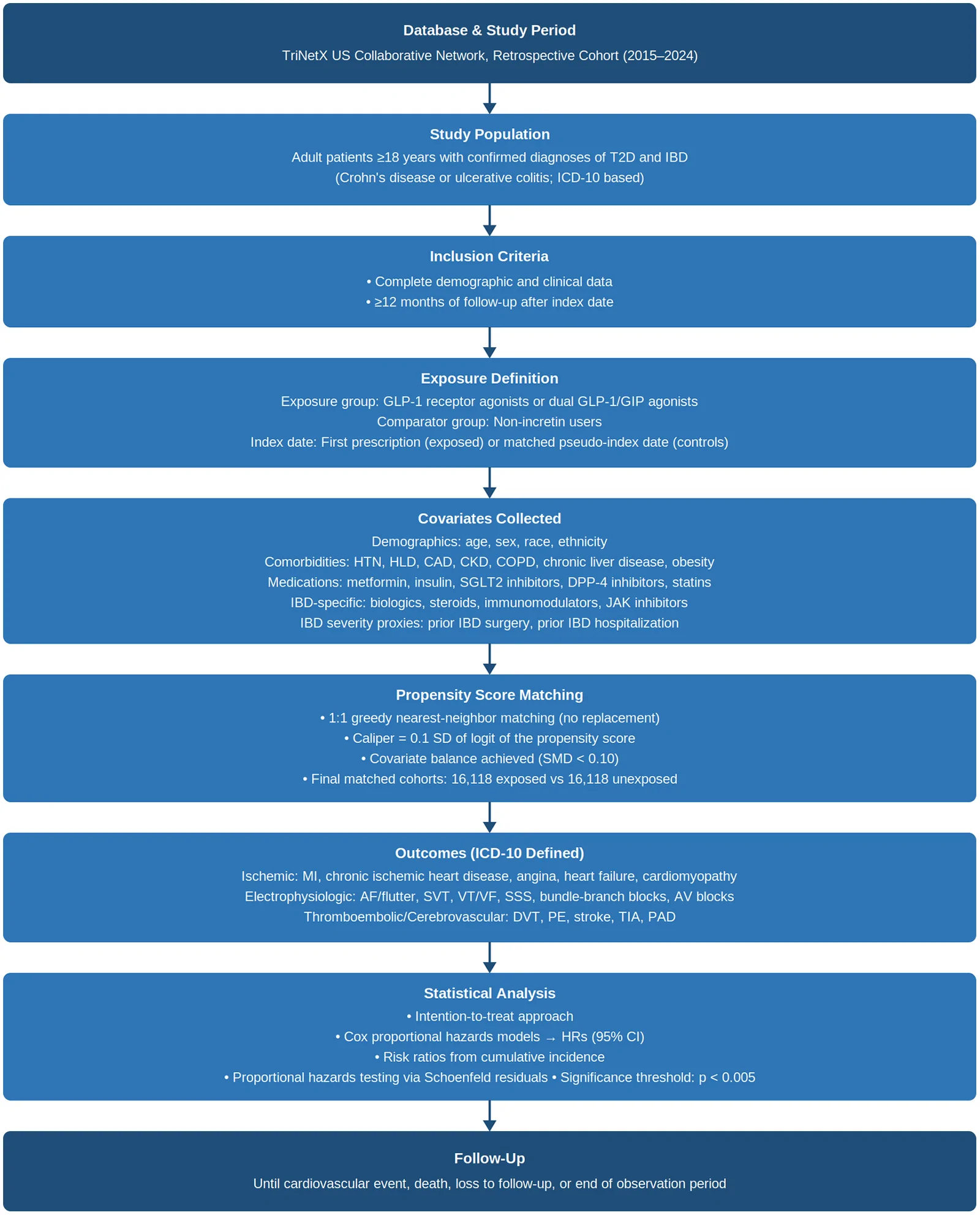
Study design and methodology flowchart.

This cohort study used data from the TriNetX database, which collects deidentified, patient-level data from electronic health records. In accordance with institutional policy and federal regulations governing research using de-identified data, Institutional Review Board (IRB) review was not required for this study. Information in the TriNetX database comes from health care organizations (HCOs), typically academic health care centers, that collect data from their main and satellite hospitals and outpatient clinics. Available data include demographics, diagnoses (ICD-10 codes), procedures (ICD-10 PCS or CPT), medications (VA Drug Classification and RxNorm), laboratory tests (LOINC), and health care utilization records. We used the US Collaborative Network in TriNetX, which includes data from more than 100 million patients from 62 HCOs. The overall study design and methodology are summarized in Fig 1.

Adults aged ≥18 years with established diagnoses of both T2D and IBD (Crohn’s disease or ulcerative colitis), between January 2015 and December 2024, were included. Inclusion criteria: (1) confirmed diagnoses of T2D and IBD, (2) availability of complete demographic and clinical data, and (3) at least 12 months of follow-up after index date.

Covariates included in the propensity score model encompassed a comprehensive range of demographic, clinical, and pharmacologic factors. Demographic variables comprised age, sex, race, and ethnicity. Clinical covariates included hypertension, hyperlipidemia, chronic kidney disease, heart failure, coronary artery disease, obesity, COPD, cerebrovascular disease, and chronic liver disease. Baseline medication profiles (insulin, metformin, sulfonylureas, DPP-4 inhibitors, SGLT2 inhibitors) were also accounted for. To address potential confounding related to IBD severity and cardiovascular risk, IBD-specific therapies including systemic and topical glucocorticoids, immunomodulators (azathioprine, 6-mercaptopurine, methotrexate), biologic agents (anti-TNF, vedolizumab, ustekinumab), and JAK inhibitors (upadacitinib) were included. Markers of IBD burden including prior IBD-related hospitalizations, emergency visits, and history of intestinal surgery were also captured. Traditional cardiovascular medications (aspirin, antiplatelet agents, anticoagulants, statins, ACE inhibitors/ARBs, beta-blockers) were incorporated to minimize residual cardiovascular confounding.

A greedy nearest-neighbor matching algorithm without replacement and a caliper width of 0.1 standard deviations of the logit of the propensity score were used to ensure optimal covariate balance between cohorts.

Statistical Analysis: All analyses were performed on the propensity-score–matched cohort under an intention-to-treat (ITT) framework, with patients indexed on the date of first qualifying prescription (exposed) or an assigned pseudo-index date (unexposed). Balance diagnostics: Covariate balance was assessed using standardized mean differences (SMDs); SMD < 0.10 was considered acceptable. We reported SMDs for all covariates and visualized propensity score overlap pre-/post-match. Effect measures: Hazard ratios (HRs) and 95% CIs were estimated using Cox proportional hazards models with robust (sandwich) variance estimators clustered by matched pair. Risk ratios (RRs) were derived from cumulative incidences. Proportional hazards assumptions were evaluated using Schoenfeld residuals, time-interaction terms, and inspection of log(–log) survival plots. The primary alpha threshold was two-sided p < 0.05.

### 2.2. Outcomes

Our study comprehensively evaluated a broad spectrum of cardiovascular outcomes, including ischemic myocardial events, electrophysiological and conduction disorders, and thromboembolic or cerebrovascular events, using ICD-10 codes to assess their association with the exposure of interest.

#### 2.2.1. Ischemic myocardial events.

1. Myocardial Infarction (I21)
2. Chronic Ischemic Heart Disease (I25)
3. Angina Pectoris (I20)
4. Heart Failure (I50)
5. Cardiomyopathy (I42)

#### 2.2.2. Electrophysiological/Cardiac conduction disorders.

1. Atrial Fibrillation/Flutter (I48)
2. Supraventricular Tachycardia (SVT) (I47.1)
3. Sudden Cardiac Death / VTach (I47.2)
4. Ventricular Fibrillation (I49.0)
5. Sick Sinus Syndrome (I49.5)
6. Left Bundle Branch Block (I44.7)
7. Right Bundle Branch Block (I45.10)
8. AV Block (1st degree) (I44.0)
9. AV Block (2nd degree) (I44.1)
10. Complete AV Block (I44.2)

#### 2.2.3. Thromboembolic and cerebrovascular events.

1. Deep Vein Thrombosis (DVT) (I82)
2. Pulmonary Embolism (PE) (I26)
3. Peripheral Arterial Disease (PAD) (I70.3–I70.7)
4. Stroke (I63)
5. Transient Ischemic Attack (TIA) (G45)

### 2.3. Statistical analysis

Relative risks (RR) and 95% confidence intervals were calculated comparing matched cohorts. Statistical significance was defined as p < 0.05.

## 3. Results

**Fig 2:**
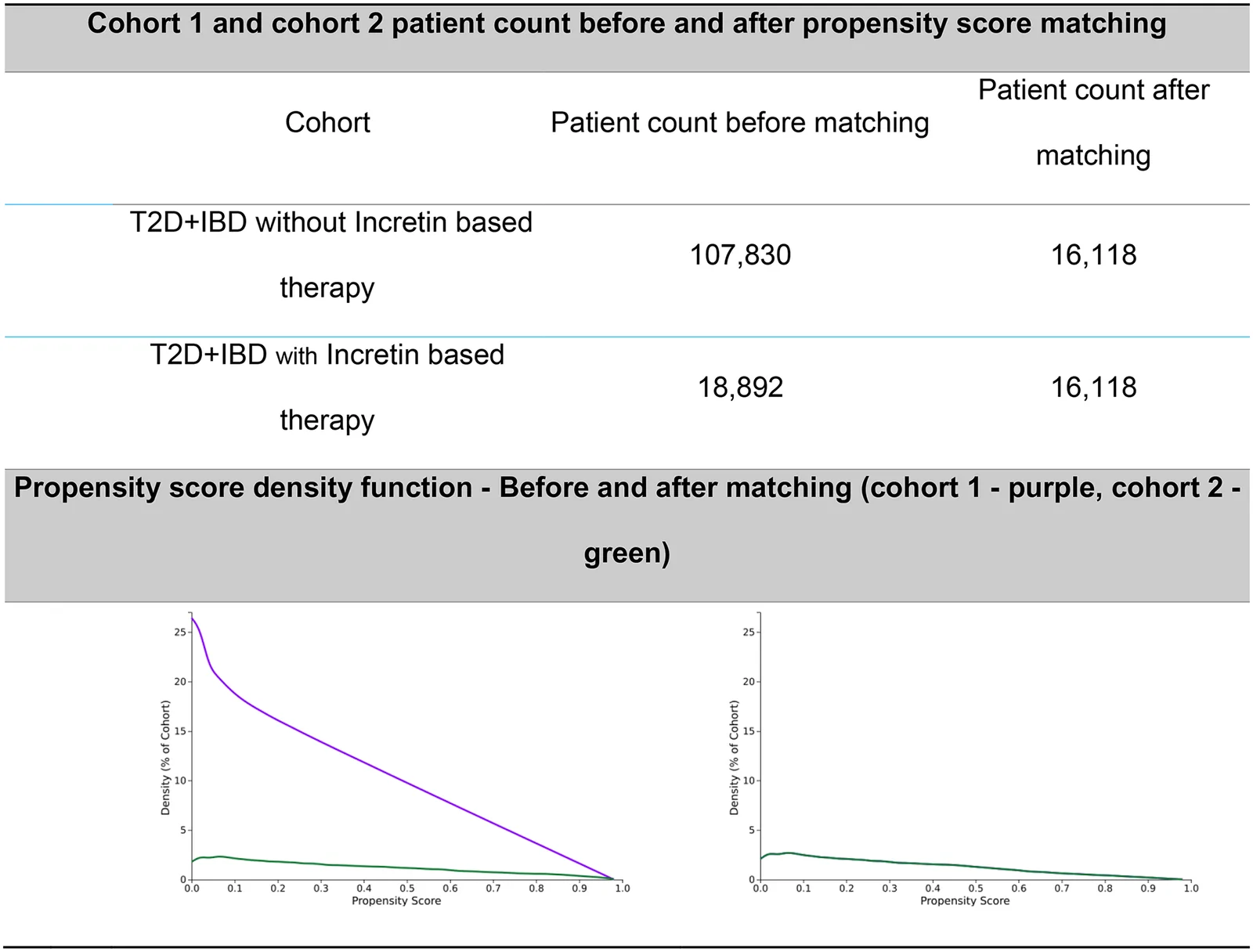
Cohort sizes and propensity score distribution before and after matching.

After matching, 16,118 patients remained in each cohort (total matched population: 32,236), with balanced baseline characteristics across all covariates (Fig 2). GLP-1 and dual GLP-1/GIP receptor agonist therapy was significantly associated with reduced risk of multiple cardiovascular outcomes (Table 1).

**Table 1:** Ischemic cardiovascular events.

Outcome	Incidence (Cohort 1)	Incidence (Cohort 2)	Hazard Ratio	HR 95% CI	Relative Risk	P-value
Myocardial Infarction	0.041	0.087	0.701	0.637–0.771	0.474	<0.05
Chronic Ischemic Heart Disease	0.231	0.297	0.936	0.896–0.978	0.776	<0.05
Angina Pectoris	0.031	0.047	0.986	0.877–1.108	0.813	<0.05
Heart Failure	0.146	0.232	0.777	0.737–0.820	0.630	<0.05
Cardiomyopathy	0.043	0.079	0.710	0.645–0.780	0.547	<0.05

### 3.1. Ischemic cardiovascular events (Fig 3)

Myocardial infarction was notably lower in the treatment group (4.0% vs. 8.2%; HR 0.701, 95% CI 0.637–0.771; RR 0.474; p < 0.05). Chronic ischemic heart disease was less frequent in GLP-1/GIP users (23.0% vs. 29.9%; HR 0.936, 95% CI 0.896–0.978; RR 0.776; p < 0.05). Cardiomyopathy showed a substantial reduction (4.3% vs. 8.0%; HR 0.710, 95% CI 0.645–0.780; RR 0.547; p < 0.05). Heart failure was significantly reduced (14.6% vs. 23.1%; HR 0.777, 95% CI 0.737–0.820; RR 0.630; p < 0.05). Angina pectoris showed a reduced incidence (3.1% vs. 4.7%; RR 0.662; p < 0.05), though the HR was not statistically significant (HR 0.986, 95% CI 0.877–1.108). As GLP-1 and dual GLP-1/GIP receptor agonists were analyzed as a combined exposure group, these associations cannot be attributed to either drug class independently. Results are summarized in Table 1.

**Fig 3:**
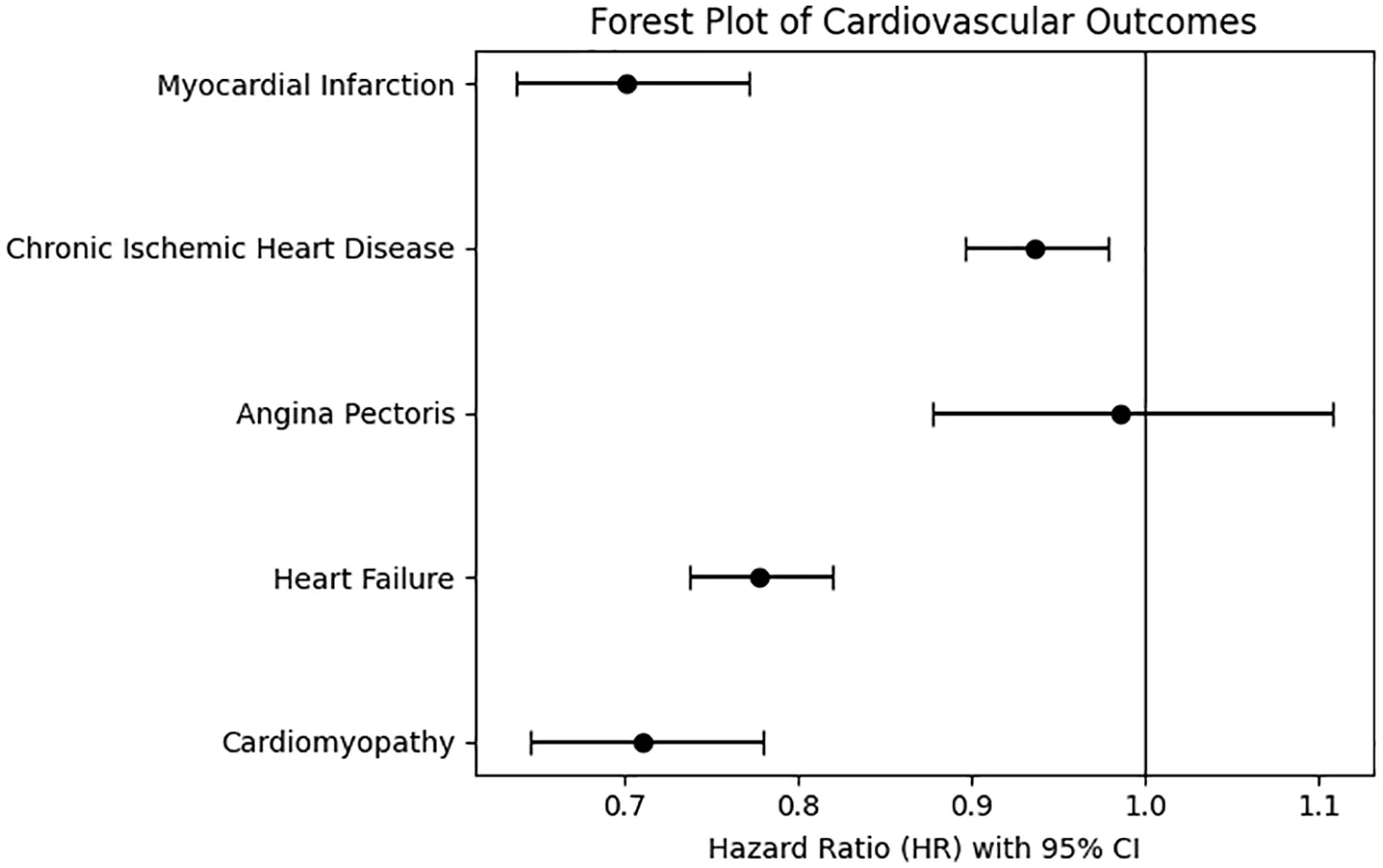
Forest plot of ischemic cardiovascular events.

### 3.2. Electrophysiological outcomes (Fig 4)

Atrial fibrillation or flutter occurred less frequently in the treatment group (11.3% vs. 16.6%; HR 0.838, 95% CI 0.788–0.890; RR 0.680; p < 0.05). Sudden cardiac death (2.0% vs. 4.1%) and ventricular fibrillation (0.2% vs. 0.6%) were significantly reduced (HR 0.694 [0.605–0.795] and HR 0.525 [0.356–0.775]; RR 0.490 and 0.394; both p < 0.05). SVT had a reduced incidence (3.9% vs. 5.7%; RR 0.693) though the HR was not significant (HR 1.017, 95% CI 0.915–1.131). Sick sinus syndrome, left and right bundle branch block showed RRs ranging 0.561–0.630, though not all HRs reached significance. AV block subtypes showed mixed results with nonsignificant HRs throughout. These results are summarized in Table 2.

**Table 2:** Electrophysiological events.

Outcome	Incidence (Cohort 1)	Incidence (Cohort 2)	Hazard Ratio	HR 95% CI	Relative Risk	P-value
Atrial Fibrillation/Flutter	0.113	0.166	0.838	0.788–0.890	0.680	<0.05
SVT	0.039	0.057	1.017	0.915–1.131	0.693	0.753
Sudden Cardiac Death	0.020	0.041	0.694	0.605–0.795	0.490	<0.05
Ventricular Fibrillation	0.002	0.006	0.525	0.356–0.775	0.394	<0.05
Sick Sinus Syndrome	0.013	0.023	0.788	0.662–0.937	0.582	<0.05
Left Bundle Branch Block	0.014	0.025	0.794	0.672–0.938	0.561	<0.05
Right Bundle Branch Block	0.028	0.045	0.961	0.850–1.086	0.630	0.520
AV Block (1st degree)	0.026	0.043	0.941	0.829–1.068	0.604	0.349
AV Block (2nd degree)	0.005	0.007	1.156	0.856–1.562	0.707	0.343
Complete AV Block	0.008	0.013	0.861	0.688–1.078	0.608	0.562

**Fig 4:**
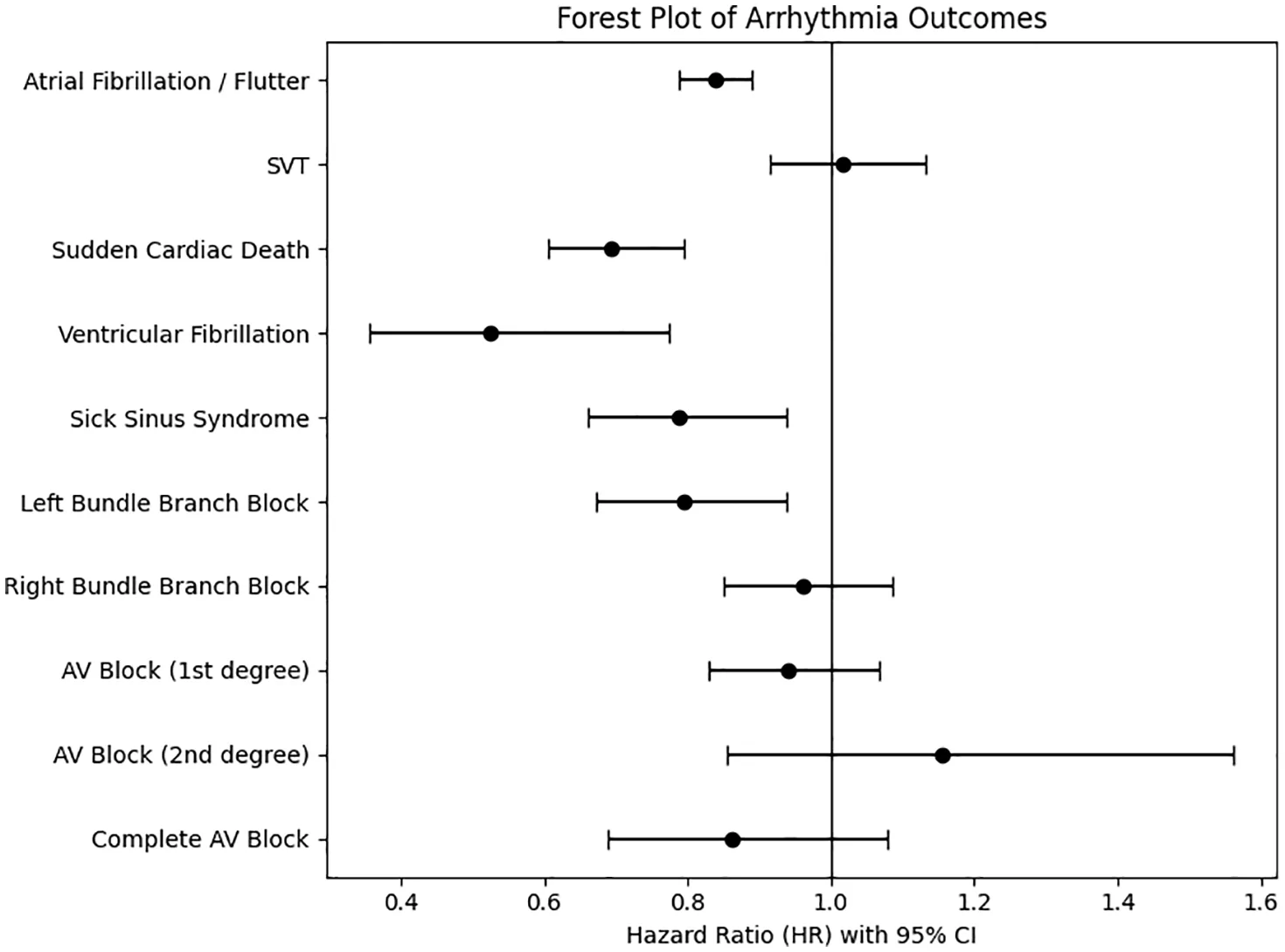
Forest plot of electrophysiological events.

### 3.3. Thromboembolic and cerebrovascular outcomes (Fig 5)

Ischemic stroke incidence was nearly halved (4.0% vs. 7.4%; HR 0.781, 95% CI 0.707–0.862; RR 0.546; p < 0.05). TIA occurred in 2.3% vs. 3.7% of controls (HR 1.00, 95% CI 0.872–1.146; RR 0.635). DVT (4.3% vs. 9.6%; HR 0.642, 95% CI 0.586–0.702; RR 0.481; p < 0.05) and PE (2.6% vs. 5.4%; HR 0.722, 95% CI 0.640–0.815; RR 0.545; p < 0.05) were both markedly reduced. PAD did not show a statistically significant difference (HR 0.991, 95% CI 0.554–1.774; p = 0.554). These results are summarized in Table 3.

**Table 3:** Thromboembolic and cerebrovascular events.

Outcome	Incidence (Cohort 1)	Incidence (Cohort 2)	Hazard Ratio	HR 95% CI	Relative Risk	P-value
Stroke (Ischemic)	0.038	0.075	0.781	0.707–0.862	0.546	<0.05
TIA	0.023	0.037	1.000	0.872–1.146	0.635	0.149
DVT	0.043	0.096	0.642	0.586–0.702	0.481	<0.05
PE	0.026	0.054	0.722	0.640–0.815	0.545	<0.05
PAD	0.001	0.002	0.991	0.554–1.774	0.556	0.554

**Fig 5:**
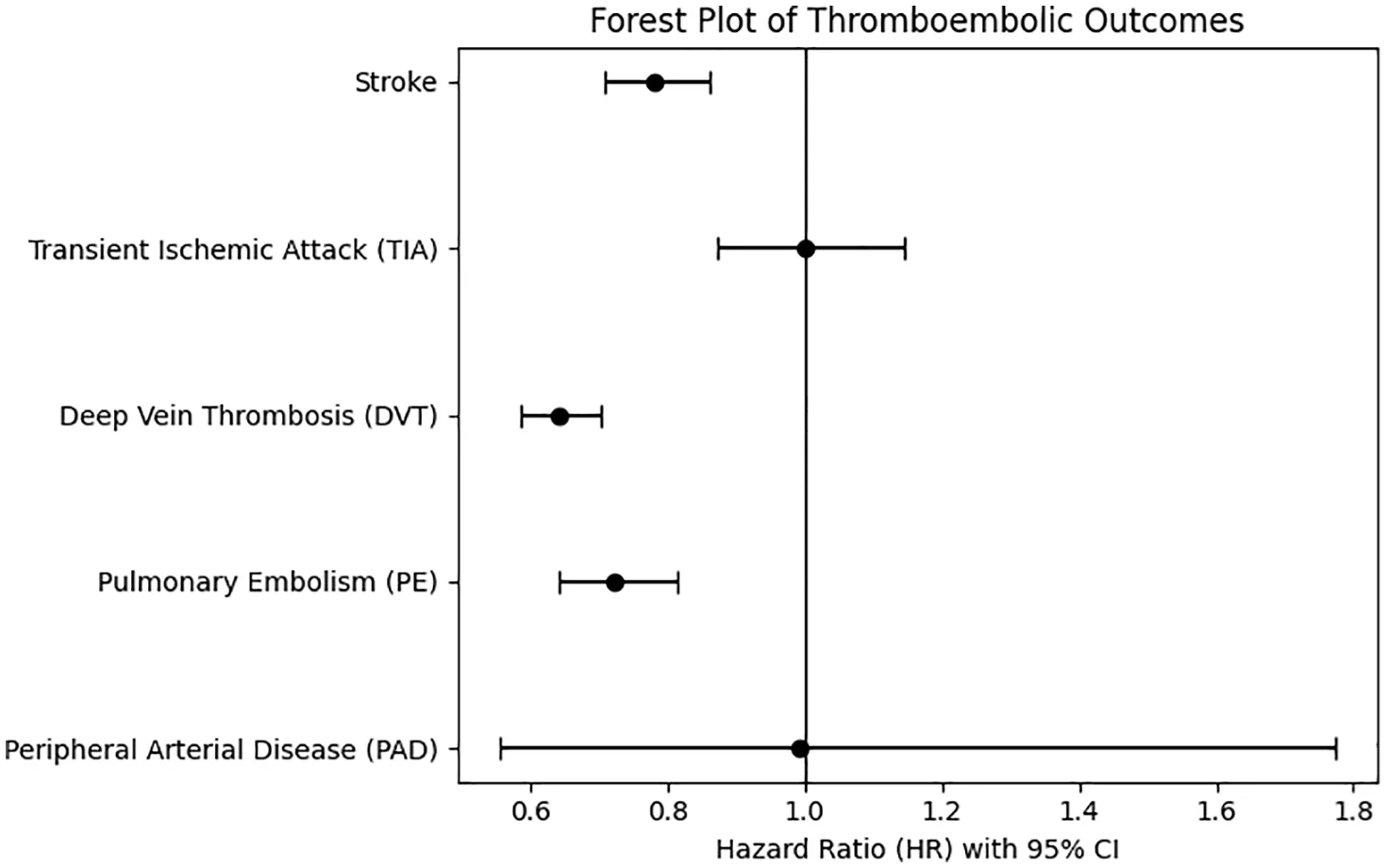
Forest plot of thromboembolic and cerebrovascular events.

## 4. Discussion

### 4.1. Cardiovascular Risk in T2D and IBD

T2D is a well-established risk factor for atherosclerotic cardiovascular disease, driven by insulin resistance, chronic hyperglycemia, and dyslipidemia, which accelerate endothelial dysfunction and atherogenesis [8]. IBD is increasingly recognized as a systemic inflammatory disorder with extravisceral manifestations, including cardiovascular morbidity [9]. The persistent systemic inflammation in IBD promotes endothelial dysfunction, arterial stiffness, and thromboembolism through upregulation of pro-inflammatory cytokines (TNF-α, IL-6), oxidative stress, and immune cell activation [10]. Patients with both T2D and IBD are doubly burdened by compounding inflammatory and metabolic insults. Future studies incorporating a comparator group of T2D patients without IBD would enable direct assessment of whether the cardiovascular benefits of incretin-based therapy differ by IBD status—a design that would complement the present findings and clarify the incremental cardiovascular burden attributable to IBD.

### 4.2. Impact of GLP-1 and dual GLP-1/GIP agonists on ischemic events

GLP-1 and dual GLP-1/GIP receptor agonist therapy was associated with significantly reduced incidence of major ischemic events, including MI, heart failure, and chronic ischemic heart disease. The incidence of MI was nearly halved (4.1% vs. 8.7%; RR 0.474; p < 0.05). This aligns with the LEADER trial demonstrating that liraglutide significantly reduced MACE in patients with T2D [11]. Similarly, SUSTAIN-6 showed that semaglutide reduced MACE [12]. Importantly, our study extends these findings to a population not represented in these landmark trials—patients with coexisting T2D and IBD. Because neither LEADER nor SUSTAIN-6 specifically enrolled patients with concurrent IBD, this analysis addresses a previously unexamined clinical context in which systemic inflammation may amplify cardiometabolic risk beyond that seen in T2D alone. However, because both drug classes were analyzed as a combined exposure group, the relative contribution of each cannot be determined from the present data.

Furthermore, GLP-1/GIP therapy was associated with a notable reduction in heart failure incidence (14.6% vs. 23.2%, RR 0.630, p < 0.05). This aligns with findings from the SUMMIT trial, which demonstrated that tirzepatide significantly reduced the risk of cardiovascular death or worsening heart failure in patients with HFpEF [13]. The potential mechanisms underlying this benefit may include improved myocardial glucose uptake, enhanced endothelial function, and attenuation of inflammatory signaling within the myocardium [29]. These mechanistic explanations are supported by preclinical data and are plausible within this observational context; however, the present study design does not allow direct testing of causality.

### 4.3. Electrophysiological protection

Patients with IBD are at increased risk for arrhythmias due to systemic inflammation and vascular changes [14]. Some studies have reported an increased incidence of arrhythmias, particularly atrial fibrillation, with GLP-1 receptor agonist use, raising concerns about potential pro-arrhythmic effects [15]. Conversely, other investigations suggest that GLP-1 therapies may exert anti-arrhythmic benefits through improved cardiac metabolism, reduced inflammation, and enhanced autonomic regulation [16].

The antiarrhythmic associations of GLP-1 and dual GLP-1/GIP agonists were also evident in our study. Atrial fibrillation/flutter occurred significantly less frequently in the treatment group (11.3% vs. 16.6%; RR 0.680; p < 0.05). This observation may be partly attributed to reductions in atrial fibrosis, oxidative stress, and sympathetic nervous system activity, as reported in preclinical studies of GLP-1 receptor activation [17]. Sudden cardiac death and ventricular fibrillation were less common among treated individuals (RRs 0.490 and 0.394, respectively). These findings are especially noteworthy given the heightened arrhythmia burden in IBD due to autonomic dysfunction, electrolyte abnormalities, and inflammation-induced myocardial remodeling.

### 4.4. Thromboembolic and cerebrovascular risk reduction

A striking observation was the marked reduction in thromboembolic and cerebrovascular events. Ischemic stroke and TIA incidences were nearly halved (RRs 0.546 and 0.635). DVT and PE were also significantly reduced (RRs 0.481 and 0.545). A large observational analysis in JAMA Network Open showed that GLP-1 receptor agonists were associated with approximately a 50% reduction in ischemic stroke and TIA risk, mediated by improvements in endothelial function and reduction in systemic inflammation [18]. CVOTs have consistently reported lower rates of ischemic stroke [19]. Regarding VTE, real-world propensity-matched data have shown significantly lower DVT and PE risks among GLP-1 users [20], though some meta-analyses suggest a potential increased DVT risk with long-term use [21]. Because GLP-1 and dual GLP-1/GIP receptor agonists were analyzed as a combined exposure in this study, class-specific contributions to these thromboembolic and cerebrovascular risk reductions cannot be determined from the available data.

### 4.5. Real-world significance and clinical implications

The cardiovascular benefits observed in this study may be partly explained by pathophysiologic features unique to patients with both T2D and IBD. Unlike traditional T2D populations, individuals with coexisting IBD experience chronic perturbations of the gut–heart axis, including impaired intestinal barrier integrity, increased translocation of microbial products such as lipopolysaccharides (LPS), dysbiosis-driven immune activation, and persistent mucosal inflammation. These processes promote systemic cytokine elevation, endothelial injury, pro-thrombotic signaling, and myocardial metabolic stress—mechanisms that accelerate atherosclerosis and increase arrhythmia susceptibility.

Emerging evidence suggests that GLP-1–based therapies may attenuate several of these IBD-specific pathways by improving barrier function, reducing LPS-mediated inflammatory signaling, modulating autonomic balance, and partially normalizing gut microbiota composition [22,30]. These mechanisms provide a biologically coherent framework for the reductions observed in our dual-disease cohort. However, these mechanistic explanations are plausible but were not directly tested in the present study, which is observational in nature and cannot establish causality between GLP-1/GIP receptor agonist use and the cardiovascular outcomes observed. Beyond glucose lowering, incretin therapies exert anti-inflammatory, anti-oxidative, and endothelial-protective effects, which are particularly relevant in settings of chronic immune activation such as IBD.

From a clinical standpoint, these findings highlight the potential role of GLP-1 and dual GLP-1/GIP receptor agonists as preferred agents in diabetic patients with coexisting IBD, offering an opportunity to address two major disease processes through a single therapeutic pathway. Integrating these agents into multidisciplinary care models—spanning endocrinology, gastroenterology, and cardiology—may meaningfully reduce morbidity, hospitalizations, and long-term cardiovascular burden.

Future research should focus on randomized controlled trials specifically designed for patients with both T2D and IBD, who remain underrepresented in current cardiovascular outcome trials. Systematic reviews, meta-analyses, and multicenter registries could further refine patient selection and support integration of incretin-based therapies into evidence-driven care frameworks for this high-risk population.

## 5. Limitations

This study has several limitations inherent to retrospective analyses of electronic health records within a federated network.

First, despite rigorous propensity score matching, residual and unmeasured confounding may persist (e.g., health behaviors, socioeconomic factors, family history, frailty), and confounding by indication or channeling bias is possible if clinicians preferentially prescribed incretin therapies to patients perceived as healthier or to those at higher cardiometabolic risk. Although measured covariates were well balanced and standardized mean differences were acceptable, propensity methods cannot account for unobserved variables or violations of positivity (limited overlap).

Second, outcome and exposure ascertainment relied on routine clinical coding. Misclassification may arise from inaccuracies or variability in ICD-10, CPT/PCS, and RxNorm/VA Drug Class coding across health care organizations. Exposure misclassification is possible since prescribing or administration records do not ensure medication adherence, dosage consistency, or persistence. Switching between antidiabetic classes and time-varying exposure could bias estimates in an ITT framework. Additionally, chronic conditions could not be reliably classified as prevalent or incident, which may influence time-to-event analyses.

Third, clinical granularity was limited. We could not systematically capture IBD activity indices (e.g., Mayo score, CDAI), endoscopic severity, or inflammatory biomarkers beyond routinely recorded values, nor could we fully assess obesity severity, tobacco exposure, or detailed lipid and blood pressure trajectories. Consequently, residual confounding by disease activity, weight loss, or lifestyle changes—each potentially on the causal pathway—remains possible.

Fourth, site-level heterogeneity (care practices, formulary access, coding accuracy) and temporal effects (e.g., COVID-19–related disruptions, evolving adoption of GLP-1/GIP) may have confounded results. The relatively recent introduction of dual GLP-1/GIP receptor agonists limits long-term follow-up. An additional comparator group of T2D patients without IBD would have enabled direct assessment of whether the observed associations differ by IBD status; this remains an important direction for future work.

Additionally, GLP-1 receptor agonists and dual GLP-1/GIP agonists were analyzed as a combined exposure group. Although this reflects real-world prescribing patterns, it may mask heterogeneity between the two drug classes. Dual agonists also have substantially shorter real-world follow-up due to their later market entry, limiting direct comparison. A dedicated head-to-head analysis of GLP-1 versus GLP-1/GIP agonists is therefore needed and is planned as future work.

Sixth, participating HCOs are predominantly large academic and tertiary institutions; results may not generalize to community or international settings. Finally, TriNetX’s data harmonization procedures, while standardized, may introduce minor discrepancies between real-time clinical data and analytic outputs. Because the platform provides aggregate-level results, investigators cannot perform independent event adjudication or apply external causal modeling beyond the built-in analytic framework.

Collectively, these limitations underscore the need for dedicated randomized controlled trials and comprehensive meta-analyses to validate causality and determine optimal treatment regimens. Where feasible within the TriNetX platform, sensitivity analyses excluding patients with documented baseline history of each endpoint (e.g., prior MI or heart failure codes before the index date) are recommended to address the prevalent versus incident outcome distinction; if not feasible, this is acknowledged as a priority for future work.

## 6. Conclusion

This observational, propensity score–matched, real-world analysis demonstrated that GLP-1 and dual GLP-1/GIP receptor agonist therapy was associated with significantly lower rates of ischemic, arrhythmic, and thromboembolic cardiovascular outcomes in patients with coexisting T2D and IBD. These associations were consistent across myocardial infarction, heart failure, atrial fibrillation, sudden cardiac death, ischemic stroke, deep vein thrombosis, and pulmonary embolism.

Several plausible mechanistic pathways may underlie these associations, including GLP-1–mediated improvements in gut barrier function, attenuation of LPS-mediated inflammatory signaling, modulation of autonomic tone, and partial normalization of gut microbiota composition. These mechanisms are biologically coherent in the context of IBD but were not directly tested in this study and should be considered hypothesis-generating.

These findings support a potential cardioprotective role for GLP-1–based therapies in patients with coexisting T2D and IBD, a high-risk population burdened by synergistic inflammatory and metabolic derangements. Further studies with longer follow-up and disease-severity adjustment are needed. The present study does not establish the comparative efficacy of the two drug classes, and the findings may be associational rather than causal. Both drug classes were analyzed as a combined exposure group. Dedicated head-to-head comparisons are required to determine whether GLP-1 and dual GLP-1/GIP receptor agonists confer differential cardiovascular benefits in this population.
